# TimeXNet: Identifying active gene sub-networks using time-course gene expression profiles

**DOI:** 10.1186/1752-0509-8-S4-S2

**Published:** 2014-12-08

**Authors:** Ashwini Patil, Kenta Nakai

**Affiliations:** 1 Human Genome Center, The Institute of Medical Science, The University of Tokyo, Tokyo, Japan

## Abstract

**Background:**

Time-course gene expression profiles are frequently used to provide insight into the changes in cellular state over time and to infer the molecular pathways involved. When combined with large-scale molecular interaction networks, such data can provide information about the dynamics of cellular response to stimulus. However, few tools are currently available to predict a single active gene sub-network from time-course gene expression profiles.

**Results:**

We introduce a tool, TimeXNet, which identifies active gene sub-networks with temporal paths using time-course gene expression profiles in the context of a weighted gene regulatory and protein-protein interaction network. TimeXNet uses a specialized form of the network flow optimization approach to identify the most probable paths connecting the genes with significant changes in expression at consecutive time intervals. TimeXNet has been extensively evaluated for its ability to predict novel regulators and their associated pathways within active gene sub-networks in the mouse innate immune response and the yeast osmotic stress response. Compared to other similar methods, TimeXNet identified up to 50% more novel regulators from independent experimental datasets. It predicted paths within a greater number of known pathways with longer overlaps (up to 7 consecutive edges) within these pathways. TimeXNet was also shown to be robust in the presence of varying amounts of noise in the molecular interaction network.

**Conclusions:**

TimeXNet is a reliable tool that can be used to study cellular response to stimuli through the identification of time-dependent active gene sub-networks in diverse biological systems. It is significantly better than other similar tools. TimeXNet is implemented in Java as a stand-alone application and supported on Linux, MS Windows and Macintosh. The output of TimeXNet can be directly viewed in Cytoscape. TimeXNet is freely available for non-commercial users.

## Background

Condition-specific gene expression profiles are used to study the response of a cell to external stimulus. Time-course gene expression profiles are especially useful in such studies since they capture the changes occurring in the cell over time. This data is often combined with protein-protein and protein-DNA interaction networks to identify sub-networks of activated genes [1]. However, many of the currently available methods that predict response networks using gene expression profiles do not incorporate the analysis of time-course data [2,3]. Instead, they give a single static response network. Some use time-based gene expression patterns to identify transcription factors activated at specific time points [4,5] to help predict the response network. Others produce static networks of genes for each time point [6,7]. Thus, most of the available methods fail to detect relationships between genes expressed at consecutive stages of the cellular response. Many also fail to identify the transient regulators that play an important role in the response but show no change in expression at the sampled time points.

We introduce a tool, TimeXNet (http://timexnet.hgc.jp/), which identifies active gene sub-networks with temporal paths using time-course gene expression profiles in the context of a weighted gene regulatory and protein-protein interaction network [8]. TimeXNet implements an algorithm that identifies the most likely paths in the network connecting genes with significant changes in expression at consecutive time intervals [8]. We show that TimeXNet is faster and more accurate at predicting active gene sub-networks than other existing tools in the study of cellular systems as diverse as the innate immune response in mouse and the yeast osmotic stress response.

## Results and discussion

### TimeXNet

**Figure 1:**
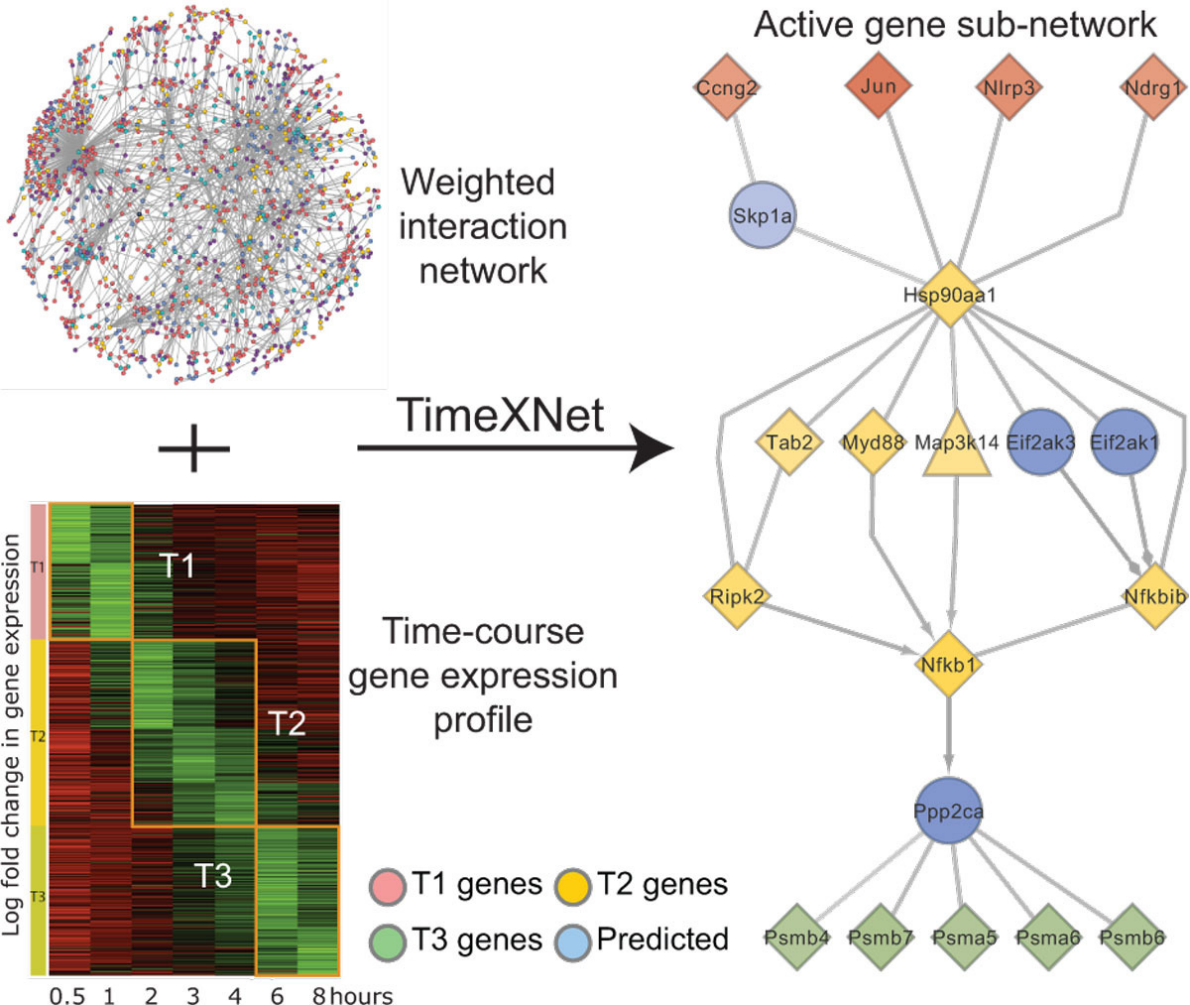
**Schematic diagram of TimeXNet algorithm**. TimeXNet identifies the most probable edges connecting genes differentially expressed at consecutive time intervals T1, T2 and T3. Node colors indicate the time of maximum fold change in gene expression. Blue nodes are genes with unknown expression predicted to act in the response network. Node shapes: diamond: up-regulation, triangle: down-regulation, circle: predicted gene.

Figure 1 shows an overview of the TimeXNet algorithm. The TimeXNet algorithm uses a large molecular interaction network, preferably containing protein-protein and protein-DNA interactions, along with post-translational modifications, weighted for reliability. It requires gene expression data collected at successive time points on cellular stimulation. The genes with significant change in expression are partitioned into three groups based on the time of their highest fold change in expression - initial response genes, intermediate regulators and late effectors. The genes in the three groups are scored according to the magnitude of their change in expression. Using the scores of the genes and the reliability weights of interactions in the starting molecular network, TimeXNet identifies the most probable paths within the interaction network connecting the initial response genes to the late effectors while incorporating the intermediate regulators. TimeXNet uses minimum-cost flow optimization to identify such paths in the network. The flow through the network is constrained so that it can only follow a time-dependent path starting from the initial response genes to the late effectors via the intermediate regulators. A detailed description of the algorithm can be found in Patil et al.[8]. TimeXNet provides the active gene sub-network in the form of a list of genes and their edges with associated flows. The flow corresponds to the importance of the gene in the sub-network and is directly proportional to its statistical significance [8]. Hence, a high flow is indicative of a high reliability of the gene included in the network as well as a significant role in the cellular response.

Thus, TimeXNet produces a single response network that incorporates the temporal information of gene expression. The network identifies paths that show the temporal relationships between genes expressed at consecutive time points. These paths also include genes that do not show change in expression levels at the sampled time points. This allows TimeXNet to identify previously unknown, transiently expressed regulators.

### Evaluation

#### Innate immune response in mouse

TimeXNet was evaluated for the identification of active gene sub-networks in the innate immune response in a weighted molecular network of 103218 interactions using gene expression data from mouse dendritic cells at 8 time points after stimulation by lipopolysaccharide (LPS). The weighted molecular interaction network was defined as a combination of mouse protein-protein and protein-DNA interactions from multiple sources including HitPredict [9], InnateDB [10], TRANSFAC [11] and KEGG [12]. Homologs of human interactions were also included. Interactions were scored using the scheme described by HitPredict [13]. The genes with more than 2 fold change in expression were assigned a score based on their relative change in expression on LPS stimulation. The immune response was classified into three consecutive time-dependent stages - early, intermediate and late. TimeXNet was used to identify the most probable paths in the molecular network between genes expressed in the early and the late phases of the immune response, incorporating genes expressed in the intervening time. The resultant network contained several new and known regulators of the innate immune response, as well as those transiently expressed between sampled time points. The predicted temporal network suggested a role for the protein phosphatase 2a catalytic subunit α in the regulation of the immunoproteasome during the late phase of the response. An analysis of time course gene expression profiles from Myd88-knockout and TRIF-knockout dendritic cells helped clarify the differences between the Myd88-dependent and TRIF-dependent pathways in the innate immune response [8].

TimeXNet was compared to two other analysis tools that use gene expression profiles to predict response networks, ResponseNet [14] and SDREM [5]. ResponseNet implements the minimum cost flow network optimization algorithm to identify paths between two groups of genes. We used ResponseNet as the baseline method, to predict the network using the initial response genes and the final effectors only, since it is unable to incorporate information from the intermediate regulators and hence cannot predict a temporal path. On the other hand, SDREM uses time course gene expression profiles to find the transcription factors functional at specific time points. It then attempts to find the paths within a molecular network connecting known regulators to these transcription factors. The performance of each tool was evaluated in terms of three properties: 1) the number of novel regulators identified from independent experimental studies [15,16], 2) the number of consecutive paths predicted that overlap with a pathway in KEGG [12], and 3) speed of execution. As can be seen in Table 1 TimeXNet identified 15-40% more novel regulators from independent experimental datasets than ResponseNet and SDREM. It also predicted paths within a greater number of associated KEGG pathways (13 compared to 2). These include the Jak-STAT signalling pathway, the Chemokine signalling pathway and the Toll-like receptor signalling pathway among others. The paths also had longer overlaps with these pathways (up to 7 consecutive, directed edges compared to 4 by SDREM). TimeXNet was also much faster than SDREM, running in minutes versus days, making it suitable for use on a local workstation. Unlike SDREM, it requires no prior knowledge of the cellular system being studied. Its ability to take the intermediate regulators into account when identifying the active paths within the network significantly improves the quality of the response network predicted.

**Table 1:** TimeXNet evaluation for the mouse innate immune response.

Method	Experimentally confirmed regulators (3 datasets)			KEGG Pathways with predicted paths (maximum length^#^)	Execution time (4 CPUs, 2.4Ghz, 12Gb RAM)	Prior knowledge required	Analysis of time-course data
**TimexNet**	49.6%^1^	69.8%^2^	54.9%^3^	13 (7 edges)	3 min	None	Yes

**ResponseNet^ǂ^**	39.2%^1^	53.5%^2^	39.2%^3^	0 (3 edges)	1 min	None	No

SDREM*	12.0%^1^	32.6%^2^	11.8%^3^	2 (4 edges)	~10 days	Initial regulatory genes	Yes

**ǂ **Results of comparison with ResponseNet taken from Patil et al.[8].

*SDREM was run with 2 starting genes (MyD88 and TRIF) and 62 target genes identified by DREM using time-course gene expression profiles.

**^# ^**Length indicates the maximum number of consecutive directed edges identified in the pathway

^1 ^Regulatory genes from Amit et al., Science, 2009

^2 ^Regulatory genes from Chevrier et al., Cell, 2011

^3 ^Target genes from Chevrier et al., Cell, 2011

#### Effect of noise in the interaction network

We evaluated the effect of noise in the interaction network on the predictions made by TimeXNet by randomly adding up to 10,000 synthetic edges (in steps of 10, 20, 50, 100, 200, 500, 1000, 2000, 5000, 10000) to the mouse interaction network and then predicting the response network. The predicted response network was evaluated for the number of known regulators identified as well as the number and extent of pathway overlap found in KEGG, as described in the previous section. We repeated this test 5 times. We found that adding random edges does not significantly affect the response network predicted by TimeXNet (Additional File 1). Similarly, randomly removing up to 10,000 edges from the mouse interaction network over 5 repetitions does not affect the predicted response network (Additional File 2). Thus, we conclude that the active gene sub-networks predicted by TimeXNet are robust to the effect of noise in the interaction network.

#### Yeast osmotic stress response

**Figure 2:**
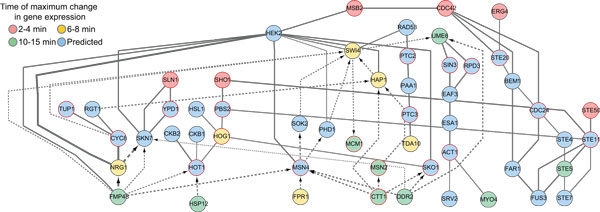
**Partial yeast osmotic stress response network predicted by TimeXNet**. Nodes indicate genes/proteins. Edges indicate the type of interaction. Solid line: protein-protein interaction, dotted line: Protein-DNA interaction. Node colors indicate time of gene expression. Blue nodes represent genes that show no significant change in expression pattern but are predicted to be part of the response network. Genes with a known role in yeast osmotic stress response are shown with a red border.

In order to evaluate the performance of TimeXNet in another species, we used it to predict the active gene sub-network in yeast in response to hyperosmotic stress [17]. We identified genes up-regulated by 1.5 fold after osmotic shock in *S. cerevisiae*. We divided the response genes into 3 groups as the initial response genes (highest fold change in expression between 2-4 minutes), intermediate regulators (highest fold change in expression between 6-8 minutes) and late effectors (highest fold change in expression between 10-15 minutes). A comprehensive yeast molecular interaction network containing 23,153 scored protein-protein and protein-DNA interactions was used [5] (personal communications, Dr. Anthony Gitter). TimeXNet was used to predict the active gene sub-network between these three groups of genes (Figure 2). TimeXNet was able to recover a significant portion of the HOG MAPK pathway, the primary response pathway to osmotic stress in yeast, including regulators, Hog1, Hot1, Pbs2, Ypd1, Msn4, Ste11 and Ste20 (See Additional File 3 for the complete list of predicted genes and interactions). The performance of SDREM and ResponseNet in predicting the yeast osmotic stress response network has been previously evaluated on this dataset [5] and we compared the predictions made by TimeXNet to those of the two methods (Table 2). Of the 30 previously known osmotic stress response genes, TimeXNet was able to identify 19 compared to 10 predicted by SDREM and 3 predicted by ResponseNet. Additionally, TimeXNet identified 5 of the 7 known transcription factors compared to 4 predicted by SDREM and 2 predicted by ResponseNet. These results indicate that TimeXNet not only successfully predicts the active gene sub-network independent of species and cellular system, but it is also superior to other tools in its prediction ability.

**Table 2:** TimeXNet evaluation for the yeast osmotic stress response

Method	Gold Standard genes^*#^	Transcription Factors^*ǂ^	Hog1
TimeXNet	19	5	Yes

ResponseNet*	3	2	No

SDREM*	10	4	Yes

^* ^Taken from Gitter et al. [5]

^# ^Out of 30 genes known to function in the yeast osmotic stress response

^ǂ ^Out of 7 transcription factors known to function in the yeast osmotic stress response

Based on the evaluation data, we conclude that TimeXNet is a significantly better tool than those currently available for the research community to analyze large amounts of time-course gene expression profiles.

### Usage

TimeXNet can be installed on the user machine by extracting the contents of a downloadable zip file. Sample data files and help files are also provided.

#### Input

The input to TimeXNet consists of the following:

*1. Three gene lists representing time-course gene expression profiles: *These gene lists represent genes that show significant changes in expression during three consecutive time intervals (initial, intermediate and late stage) on exposing the cell to external stimulus. Each gene list is given in the form of a tab-delimited file containing the gene name and the gene score. The gene score represents the value assigned to the gene based on its change in expression, usually the log fold change. The three gene groups are mutually exclusive i.e. genes in one group cannot be present in another.

*2. Weighted interaction network: *The interaction network is also given as a tab-delimited file containing the each edge denoted by two genes, the type of interaction (unidirectional/bidirectional) and its reliability score. The edges may denote either a physical or functional association between two genes/proteins. The score indicates how reliable the edge is based on experimental or genomic annotation information and should be between 0 and 1. TimeXNet provides a comprehensive network of weighted protein-protein, protein-DNA interactions and post-translational modifications in mouse.

*3. Algorithm parameters: *These include two real positive constants, γ1 (gamma1) and γ2 (gamma2), which are used to decide the number of initial response genes and intermediate regulators to be included in the predicted response network. TimeXNet requires the GNU Linear Programming Kit (GLPK) in order to solve the optimization problem. It tries to automatically detect installed GLPK. If it fails to find a local copy of the GLPK, it requests the user to install it and provide the location to TimeXNet.

*4. Output location: *TimeXNet generates several output files and requires the user to specify the output directory where these files will be stored.

#### Execution

**Figure 3:**
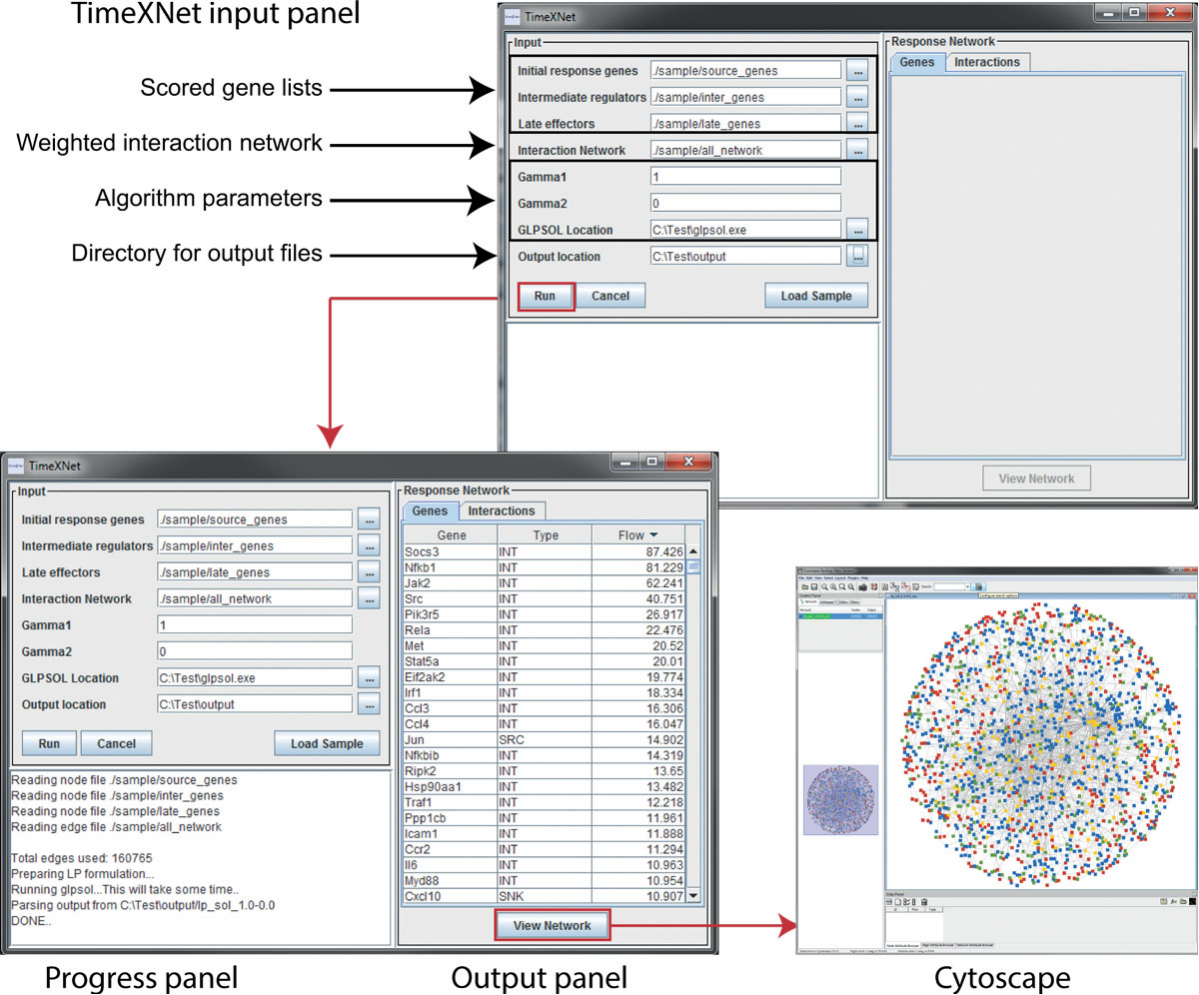
**TimeXNet user interface**. The TimeXNet user interface consists of three main panels - the input panel, the progress panel and the output panel. TimeXNet requires three scored gene lists, a weighted interaction network and two real positive constants in order to run. Sample data for evaluation can be loaded into the user interface using the "Load Sample" button. During execution, TimeXNet updates the progress panel to inform the user of the current status. After completion, the tables in the output panel are populated with the genes and interactions in the predicted response network along with their type and flow. The "View Network" button invokes Cytoscape to show the response network predicted by TimeXNet.

After extracting all the files from the downloaded zip file, TimeXNet can be run in three modes:

*1. User Interface: *TimeXNet can be run through a user-interface (Figure 3) by double-clicking the timexnet.jar file. The user interface also allows the user to load sample data and network for analysis. On execution, TimeXNet displays the identified genes and interactions of the predicted response network in a tabular format on the user interface. The predicted response network can also be viewed in Cytoscape, which is launched directly from TimeXNet. The results are stored in several tab-delimited files at the specified location on the user's machine.

*2. Command line: *TimeXNet can also be run from command line with the same input parameters as those of the user interface. This version is particularly useful for running TimeXNet on a supercomputer. The predicted response network is saved in the form of tab-delimited files at the specified location.

*3. Iterative command line: *The command line version of TimeXNet can be run in an iterative manner for a range of γ1 and γ2 values in order to identify the combination that results in an optimal network i.e. one with the highest number of genes from the three groups and the fewest number of low reliability edges from the starting network. To be run in this mode, TimeXNet requires a range of real positive values for γ1 and γ2. The output provides a statistics file containing the number of original genes and low reliability edges in the predicted network for all combinations of γ1 and γ2 run by TimeXNet. A separate directory is created at the user-specified output location for each combination of γ1 and γ2 values. The predicted response network for each γ1 and γ2 combination is saved in the form of tab-delimited files similar to the single command line and user interface version.

#### Output

All versions of TimeXNet create and store tab-delimited files containing the genes and interactions of the predicted sub-network along with their flows in a specified location. These can be directly uploaded into Cytoscape. Genes in the predicted response network are assigned a type- SRC, INT, SNK and NOD. Genes of type SRC are a subset of the initial response genes given to TimeXNet as input. INT genes are a subset of the intermediate regulators, while SNK genes are part of the final effectors showing large changes in expression at the final time points. The NOD genes are those that do not show change in expression at the sampled time points but are predicted by TimeXNet to be a part of the response network. Additionally, the formulation of the optimization problem given as input to the GLPK to predict the response network and the final edge list used to generate the optimization problem are also stored, along with the unformatted solution. Finally, a log file showing the detailed progress of the TimeXNet run including a list of duplicate edges ignored, edges and nodes with erroneous scores, and the detailed output of the GLPK is stored in the output directory.

Additional details about installation, input-output files and formats, and usage of TimeXNet can be found at http://timexnet.hgc.jp/.

## Conclusions

TimeXNet is a fast and accurate method to identify active gene sub-networks using time-course gene expression profiles. It produces a single response network of genes showing differential expression at consecutive time points with each gene/node and interaction/edge scored for its potential importance in the predicted response network. TimeXNet does not require any starting knowledge of the response pathway being studied. It is able to identify transiently expressed regulators or those showing no change in expression using the time-course gene expression profiles. This allows the user to identify previously unknown regulators. Thus, TimeXNet helps towards a greater understanding of the temporal relationships between regulators of cellular events. The current version of TimeXNet can only find relationships between three groups of genes. Future versions will be capable of working with a larger number of gene groups as well as incorporating other forms of information such as levels of protein phosphorylation.

## Methods

### Implementation

TimeXNet is implemented in Java as a stand-alone application in a format compatible with Linux, Windows and Macintosh. It requires the Java Runtime Environment 1.7 and the GNU Linear Programming Kit (GLPK) to be installed on the user's machine. Both are freely available and easy to install. TimeXNet looks for an existing copy of GLPK. If it is not found, TimeXNet requests the user to install GLPK from an installable included in the zip file. The predicted response network can be viewed as a table or a network in Cytoscape [18], which is bundled with TimeXNet. The network is formatted such that the temporal expression patterns of the genes are clearly visible.

### Availability and requirements

**Project name: **TimeXNet

**Project home page: **http://timexnet.hgc.jp/

**Operating system(s): **Platform independent.

**Programming languages: **Java.

**Other requirements: **GNU Linear Programming Kit

**License: **Free to non-commercial users

## Competing interests

The authors declare that they have no competing interests.

## Authors' contributions

AP designed and implemented the algorithm and software, evaluated results and wrote the manuscript. KN evaluated results, provided critical comments on the manuscript and contributed computational resources.

## Supplementary Material

Additional file 1 **(DOCX): Performance of TimeXNet to successive addition of random edges to the initial interaction network**. a) Known targets and regulators identified from Amit et al. and Chevrier et al., b) Pathways with up to 3 consecutive edges and the maximum length of overlapping path predicted by TimeXNet in the response network predicted from an interaction network with 10, 20, 50, 100, 200, 500, 1000, 2000, 5000, 10000 random edges added. Click here for file

Additional file 2 **(DOCX): Performance of TimeXNet to successive removal of random edges to the initial interaction network**. a) Known targets and regulators identified from Amit et al. and Chevrier et al., b) Pathways with up to 3 consecutive edges and the maximum length of overlapping path predicted by TimeXNet in the response network predicted from a basal interaction network with 10, 20, 50, 100, 200, 500, 1000, 2000, 5000, 10000 random edges removed. Click here for file

Additional file 3 **(XLSX): List of genes predicted by TimeXNet in the yeast osmotic stress response network**. TimeXNet was run with gamma1 = 0.5 and gamma2 = 0.5 after testing all combination from 0 to 5 for the optimal network. Click here for file

## Declaration

This work was partially supported by the Cabinet Office, Government of Japan and JSPS through the Funding Program for World-Leading Innovative R&D on Science and Technology (FIRST Program). The publication charges for this article were funded by the Grant-in-Aid for Young Scientists by the Japan Society for the Promotion of Science to AP.

This article has been published as part of *BMC Systems Biology *Volume 8 Supplement 4, 2014: Thirteenth International Conference on Bioinformatics (InCoB2014): Systems Biology. The full contents of the supplement are available online at http://www.biomedcentral.com/bmcsystbiol/supplements/8/S4.
