# Supplementary material for: TimeXNet: Identifying active gene sub-networks using time-course gene expression profiles
Source: BMC Syst Biol. 2014 Dec 8;8(Suppl 4):S2. doi: 10.1186/1752-0509-8-S4-S2 (PMC4290689; doi:10.1186/1752-0509-8-S4-S2)
Supplement: Additional file 1 — (DOCX): Performance of TimeXNet to successive addition of random edges to the initial interaction network. a) Known targets and regulators identified from Amit et al. and Chevrier et al., b) Pathways with up to 3 consecutive edges and the maximum length of overlapping path predicted by TimeXNet in the response network predicted from an interaction network with 10, 20, 50, 100, 200, 500, 1000, 2000, 5000, 10000 random edges added. [file 1752-0509-8-S4-S2-S1.docx]

a)
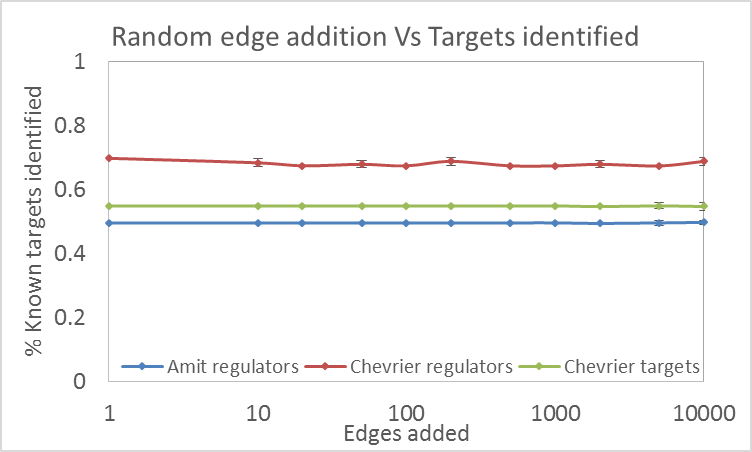

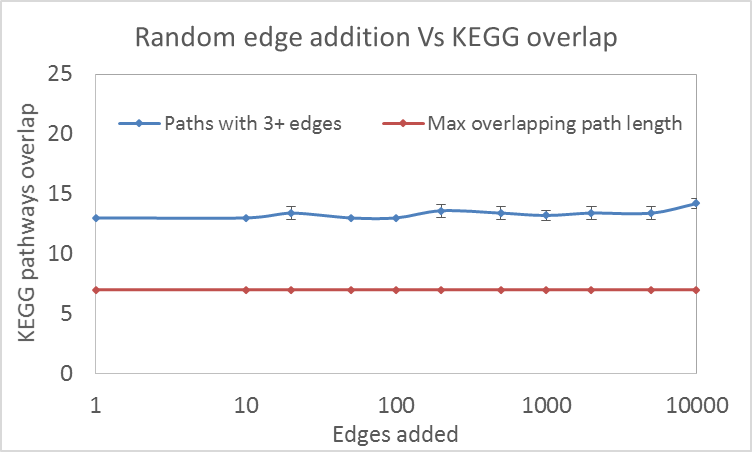


b)

**Performance of TimeXNet to successive addition of random edges to the initial interaction network.** a) Known targets and regulators identified from Amit et al. and Chevrier et al., b) Pathways with up to 3 consecutive edges and the maximum length of overlapping path predicted by TimeXNet in the response network predicted from an interaction network with 10, 20, 50, 100, 200, 500, 1000, 2000, 5000, 10000 random edges added.
